# Older People’s Perceptions of Pedestrian Friendliness and Traffic Safety: An Experiment Using Computer-Simulated Walking Environments

**DOI:** 10.3390/ijerph120810066

**Published:** 2015-08-21

**Authors:** Daniela Kahlert, Wolfgang Schlicht

**Affiliations:** Exercise and Health Science, Stuttgart Research Initiative Human Factors in Ageing, Technology, and Environment, University of Stuttgart, Nobelstr. 15, Stuttgart 70569, Germany; E-Mail: wolfgang.schlicht@inspo.uni-stuttgart.de

**Keywords:** physical activity, walking for recreation, built environment, older people, computer-simulated experiment

## Abstract

Traffic safety and pedestrian friendliness are considered to be important conditions for older people’s motivation to walk through their environment. This study uses an experimental study design with computer-simulated living environments to investigate the effect of micro-scale environmental factors (parking spaces and green verges with trees) on older people’s perceptions of both motivational antecedents (dependent variables). Seventy-four consecutively recruited older people were randomly assigned watching one of two scenarios (independent variable) on a computer screen. The scenarios simulated a stroll on a sidewalk, as it is ‘typical’ for a German city. In version ‘A,’ the subjects take a fictive walk on a sidewalk where a number of cars are parked partially on it. In version ‘B’, cars are in parking spaces separated from the sidewalk by grass verges and trees. Subjects assessed their impressions of both dependent variables. A multivariate analysis of covariance showed that subjects’ ratings on perceived traffic safety and pedestrian friendliness were higher for Version ‘B’ compared to version ‘A’. Cohen’s d indicates medium (d = 0.73) and large (d = 1.23) effect sizes for traffic safety and pedestrian friendliness, respectively. The study suggests that elements of the built environment might affect motivational antecedents of older people’s walking behavior.

## 1. Introduction

Physical activity reduces the risk of premature death and disease and promotes health, well-being and functional independence in old age [1,2]. However, despite the positive effects of physical activity, only a small number of people are physically active, and older adults in particular fail to get the recommended daily amount of physical activity [3,4]. This issue is of particular relevance in populations in which the proportion of older adults is increasing and ageing is associated with morbidity, which describes most populations in Europe [5]. Therefore, motivating older people to increase their physical activity is of high importance.

Among older adults, walking is a common and preferred physical activity [6]. Older people who walk regularly have fewer depressive symptoms and are less functionally impaired [7,8]. Moreover, low-intensity activities, such as walking, have been shown to reduce the risk of all-cause mortality, cardiovascular mortality and death by other causes [9]. 

Socio-ecological models [10] and environmental gerontology approaches [11] postulate that elements of the built environment affect older people’s behavior because such elements create ‘opportunity structures’ that may increase or decrease the likelihood of being physically active in a given living environment. Older people spend more time in their living environments and prefer to age in the environments in which they lived as younger adults [12]. 

Many studies have investigated the relationship between environmental characteristics and older people’s walking behavior [13,14]. However, neither the narrative reviews nor the meta-analyses of these studies reveal any clear overall pattern. Rather, the association between environmental features and specific types of physical activity, such as recreational walking, remains ambiguous [13]. Yen and colleagues recently developed a program theory based on the results of a realist synthesis, which is a theory-driven interpretive method of evidence synthesis [14]. Their program theory identifies three interrelated environmental contextual factors that influence people’s readiness and motivation to engage in daily physical activity: connectivity of streets, land use mix and environmental aesthetics. The authors also stressed the importance of people’s subjective impressions of environmental safety (*i.e.*, traffic- and crime-related safety), which they found to be a key moderating mechanism between environmental contextual factors and older people’s decisions to remain or become active [14].

*Connectivity* and *land use* are macro-scale environmental factors and thus are often difficult to modify quickly in existing living environments. Accordingly, these environmental factors are relevant only to long-term public planning considerations. In contrast, *aesthetics* and *safety,* the other two elements of the program theory developed by Yen *et al*. [14], are micro-scale environmental factors. Micro-scale environmental factors, which include street lighting, pedestrian crossings, the availability and quality of sidewalks and the presence of green vegetation, are easier to change in the short term.

Studies regarding the influence of micro-scale environmental factors on physical activity have yielded inconsistent results [15], which could be due to a number of different factors. For example, there is no clear definition of how far the “living environment” extends [16] and thus different studies have applied different definitions, which has likely led to different outcomes. In addition, most existing studies were conducted using written questionnaires, such as the Neighborhood Environment Walkability Scale (NEWS) questionnaire [17] that ask people to state their impressions of environmental attributes based on written descriptions. Because environmental attributes co-occur differently for different study participants, both within and between studies, ‘spill-over effects’ may occur. The co-occurrence of environmental characteristics makes it difficult to differentiate the impact of a single environmental factor [18]. Another problem with questionnaires is that subjects are not involved in outdoor environments when they respond to questions but rather must rely on written descriptions of such settings. 

The use of panoramic photographs is one method of increasing participants’ awareness of the environments of interest and enhancing their environmental involvement [19,20,21]. In a study conducted by Van Cauwenberg *et al*. [21], participants had to evaluate the ‘invitingness’ of environments depicted in photographs (forced choice). In a study conducted by Mertens *et al*. [19], participants looked at photographs in which various micro-scale environmental factors (e.g., speed bumps, general upkeep, evenness of the cycling path, vegetation) had been manipulated. Subjects in the Mertens *et al*. [19] and Van Cauwenberg *et al*. [21] studies were asked to consider and rate their motivation to use bicycles for transportation and to walk for transportation, respectively, based on these photographs. However, photographs provide static impressions of the living environment [19,20]. In contrast, computer graphic simulations provide a dynamic perceptual experience [20,22].

This study aims to stimulate the research field by applying a method that uses computer-simulated living environments. The variables were based on Yen’s program theory [14]. The study reported here investigated the impact of micro-scale environmental features (*i.e.*, the separation of parked cars and roads from sidewalks by grass verges) on older people’s perceptions of traffic safety and pedestrian friendliness of a particular living environment. 

### Hypothesis

It was hypothesized that a computer simulated stroll through a sidewalk bounded by grass verges and cars separated in parking spaces will lead subjects to higher ratings of their perception of safety and pedestrian friendliness compared to sidewalks partially blocked by cars. 

## 2. Experimental Section 

The study was based on an experimental, randomized research design that uses two versions of a simulated living environment. Using randomized allocation, participants were assigned to watch one of two videos representing a six-minute recreational walk through a typical German living environment. 

### 2.1. Sample

Seventy-four older people identified through associations, clubs and organizations (e.g., music society) in the city were recruited consecutively to the study. The required sample size was determined using an *a priori* G*Power calculation assuming medium effect sizes (n = 68 for MANOVA with special effects and interactions in the first step and n = 72 for ‘protected’ t-tests of independent group means in the second step; *p* < 0.05; Power 0.80). Subjects were eligible if they met all of the inclusion criteria, specifically, they were (a) 60 years of age or older, (b) retired, (c) able to walk, (d) living an autonomous life, and (e) residing in an urban environment. Subjects were excluded if major functional limitations hindered their walking ability. The inclusion criteria were assessed using a questionnaire, and functional limitations were assessed using the German version of the 36-item Health Survey [23]. Specifically, the 10-item subscale of the short form, called ‘physical functioning’, was used for this purpose.

Sample characteristics are summarized in Table 1. The average age was 71.1 years (SD = 6.16), and ages ranged from 60 to 86 years. Gender distribution was not balanced (43.2% women; 56.8% men). All study participants took part voluntarily and without compensation and provided full informed consent prior to participation. 

**Table 1:** Descriptive statistics of study participants and dependent variables.

Variable	Sample	“Version A”	“Version B”
Age Means (SD)	71.1 (6.1)	71.6 (6.4)	70.5 (5.9)
Women (%)	43.2	45.9	40.5
Grown up in Germany (%)	100	100	100
Education (%)			
Secondary modern school (Hauptschule)	18.9	24.3	13.5
Intermediate modern school (Realschule)	52.7	40.5	64.8
Secondary/ High school (Gymnasium)	28.4	35.2	21.7
Physical activity			
Moderate-to-vigorous PA in min/week	160.78	156.02	165.27
Means (SD)	(182.39)	(196.74)	(170.43)

### 2.2. Protocol and Study Design

All study participants who fulfilled the inclusion criteria were invited to the University of Stuttgart campus, where a special room had been prepared for the study. A research assistant welcomed participants as they arrived and obtained informed consents and declarations of voluntary participation. Thereafter, participants completed a questionnaire related to their demographic backgrounds and usual amounts of physical activity.

Afterward, participants were directed to carefully read the following instructions:

You are now going to watch a video that is approximately six minutes long. The video shows a stroll through a typical German city. Please imagine that you are walking through the city without any specific purpose. The weather is nice, not too hot and not too cold, the sun is shining, and there is no strong wind. You are feeling well and you don’t have any problems that hinder you from walking.

After watching the video, you will be asked to complete a questionnaire about your perceptions and impressions during your stroll through the city. This will not be a knowledge test. If you have any questions at this point, please contact the investigator. Afterwards, the video will start.

**Figure 1:**
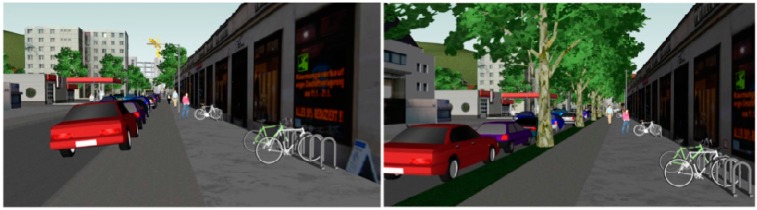
Example of the same route section in Version A and Version B of the computer simulated living environment.

Both videos showed a stroll through a living environment in a typical German city. Depending on their randomized assignment, each subject saw either Version A or B of the video. Both show the same walking route, but Version A depicts a typical city and living environment in which (some) cars are parked such that they partially cover the sidewalk, whereas version B depicts a living environment in which cars are parked in parking spaces adjacent to the sidewalk and a grass verge with trees is between the sidewalk and the parking spaces (see Figure 1). No other features differentiate the environments shown in the two videos. For instance, the number of cars, sidewalk width and number of fictitious pedestrians are the same. 

**Figure 2:**
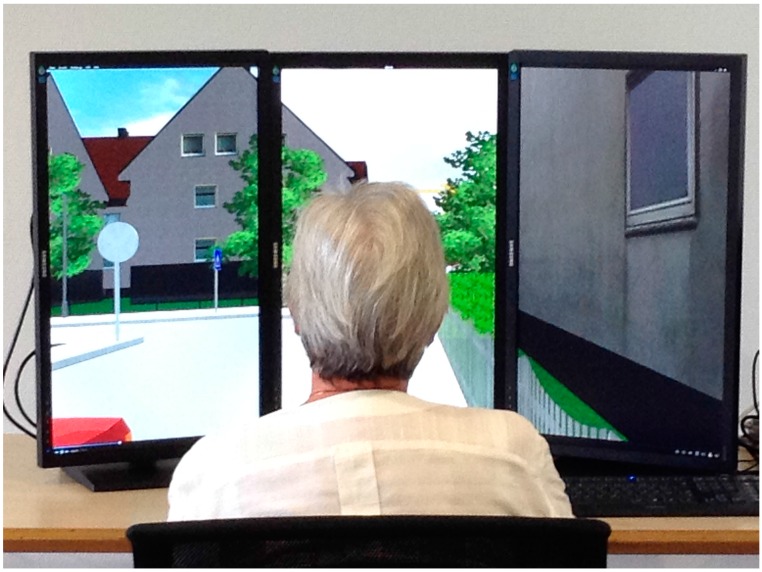
Picture of the laboratory setting.

The six-minute videos were recorded using Walkabout3d software and movie-capture tool Dxtory. They were presented on three 27-inch full-HD screens with resolutions of 3,466 × 1,920 pixels. The three screens were merged into one viewing station and study participants were seated in front of it (see Figure 2).

After the video was finished, subjects rated their impressions of traffic safety and pedestrian friendliness using a 7-point Likert scale (see below for additional details regarding measurements). The investigator then thanked the subjects and answered any questions.

### 2.3. Development of the Simulated Living Environment

The living environments presented in the videos were designed using Google SketchUp Pro 2014 and 3D Warehouse software. SketchUp Pro is used to build 3D environmental models, and 3D Warehouse is a database containing several replicas (e.g., houses, trees) that can be used and integrated in Google SketchUp Pro. The simulated city included typical areas (e.g., the inner city, residential neighborhoods, schools, shops) and common elements, including street furniture (e.g., traffic lights, cross-walks), pedestrians and cars. The dimensions and proportions of the simulated city followed official guidelines, including German roadwork guidelines (which relate to, e.g., the widths of sidewalks and crosswalks). 

Three citizens independently assessed the realism of the first version of the environmental model (e.g., to identify missing elements of a typical German city, such as bicycles). After their feedback was implemented, five researchers and two additional citizens independently judged the second version of the model, in which they mainly focused on scale relation between the elements of the simulated city (e.g., size of cars compared with height of people). This process led to the final version of the basic model (Version A), which depicts a living environment with (some) cars parked partially on the sidewalk, which is very common in German cities. Version B was identical to Version A except for the experimentally manipulated changes described above.

### 2.4. Measurements 

#### 2.4.1. Socio-Demographic Characteristics

Age, sex and educational level were assessed with a standardized questionnaire. In addition, subjects were asked whether they grew up in Germany and, if not, how long they had lived in Germany. The responses revealed that all subjects grew up in Germany, which ensured that all of them were familiar with the type of city and living environment simulated in the videos. 

#### 2.4.2. Physical Activity

Participants were asked, using a single item, how many minutes per week they are moderately active, in general: “How many minutes per week do you engage in physical activity with moderate intensity (*i.e.*, you can talk but not sing during the activity you perform).”

#### 2.4.3. Dependent Variables 

Questions related to the two dependent variables, ‘traffic safety’ and ‘pedestrian friendliness’, were extracted from existing questionnaires and audit tools (e.g., the German version of NEWS: [24]; the Systematic Pedestrian and Cycling Environmental Scan instrument: [25]; the Physical Activity Neighborhood Environment Survey: [26]).

*Traffic safety.* A four-item scale was used to measure subjects’ impressions of traffic safety (Table 2). Each subject rated each item (e.g., “The traffic in this city makes it hard for pedestrians to walk around”) on a seven-point Likert scale (1 = *not at all true* to 7 = *completely true*). Scale results were calculated as mean scores, and the scale showed sufficient internal consistency (Cronbach’s α = 0.70).

*Pedestrian friendliness.* An eight-item scale was used to assess subjects’ impressions of pedestrian friendliness (see Table 1). Subjects were asked to rate each item (e.g., “I would like to walk around in this city”) on a seven-point Likert scale (1 = *not at all true* to 7 = *completely true*). Scale results were calculated as mean scores, and the scale showed high internal consistency (Cronbach’s α = 0.93).

**Table 2:** Items to assess traffic safety and pedestrian friendliness.

Traffic Safety
The traffic in this city makes it hard for pedestrians to walk around.
I feel safe in this city walking around.
The street crossings support pedestrians.
I am confident being as a pedestrian on the sidewalk.
**Pedestrian Friendliness**
I feel comfortable being as a pedestrian in this city.
The sidewalks in this city are well maintained.
This city makes it easy to walk around.
This city is inviting for pedestrians.
I would love to walk around in this city.
It is interesting to walk around in this city.
This city is pleasant for pedestrians.
I would like to be a pedestrian in this city.

#### 2.4.4. Analysis

A multivariate analysis of covariance (MANCOVA) was conducted in the first step. Gender and physical activity were controlled reflecting their potential to influence perceptions of traffic safety and pedestrian friendliness. In order to determine the occurrence of group differences, an alpha error accumulation protected t-test was conducted in the second step.

## 3. Results 

The sample statistics are depicted in Table 1. Table 3 contains data for the two subsamples (Version A *vs*. Version B × dependent variables). Distribution of age, gender, education and amount of physical activity did not differ between the two groups (*p* > 0.05).

In a first step, a multivariate analysis of covariance showed that the experimental manipulation significantly affected both *pedestrian friendliness* and *traffic safety, F* (2, 68) = 12.91, *p* < 0.01, Eta^2^ = 0.27. The analysis controlled for subjects’ gender and amount of physical activity, neither of which showed significant effects (*p* > 0.05). In the second step, two t-tests for the independent samples revealed statistically significant differences in subjects’ ratings of *traffic safety* and *pedestrian friendliness* (see Table 3). In accordance with the hypothesis, subjects who viewed Version B of the video judged the simulated environment as safer than subjects who watched Version A did, *t*_(65)_ = 3.15, *p* < 0.01, *d* = 0.73. A similar result was found for *pedestrian friendliness*. Specifically, subjects who watched version B perceived the simulated environment as more pedestrian friendly than subjects who watched version A did, *t*_(65)_ = 5.26, *p* < 0.01, *d* = 1.23. Cohen’s d indicates medium (d = 0.73) and large (d = 1.23) effect sizes for traffic safety and pedestrian friendliness, respectively.

**Table 3:** Mean values, SD and score statistics for dependent variables (t-tests for independent group means).

Group	Dependent Variable	*n*	*M (SD)*	*t (df)*	*p*	*d*
Version A Version B	Traffic safety	37 37	4.61 (1.28) 5.43 (0.92)	3.15 (72)	<0.01	0.73
Version A Version B	Pedestrian friendliness	37 37	3.56 (1.41) 5.07 (1.00)	5.26 (72)	<0.01	1.23

## 4. Discussion

This study addressed whether micro-environmental changes affect older people’s perceptions of traffic safety and pedestrian friendliness. Specifically, it was hypothesized that a combination of parking spaces and grass verges with trees would increase older people’s perceptions of traffic safety and pedestrian friendliness of a living environment.

The results of the experiment involving 74 retired people living in a German urban environment confirmed our hypothesis. Grass verges with trees and a clear separation of parking spaces from the sidewalk increased older adults’ perceptions of pedestrian friendliness and traffic safety (related to automobile traffic). The latter is a key mechanism for motivating people to be active in their living environments [14]. In addition, grass verges and a separation of cars from the sidewalk led to higher ratings of pedestrian friendliness, which could be considered a proxy for (older) people’s decisions to be physically active. The data support the general assumption that people living in safe, pedestrian-friendly environments may be more active than those living in environments in which vehicles are parked on the sidewalk. It is important to note that we did not measure the effect of people’s living environment upon their physical activity but measured proxy variables to assess perceptions of attributes, which could impact upon older people’s motivation to walk [14].

The distinctive feature of the present study that extends the existing literature in this field is its experimental research design, which used computer-simulated dynamic living environments. It has been suggested that virtual color videos provide a realistic mode of presentation and have the potential to improve internal validity through experimental variation [20,22]. A recent study by Nasar *et al*. [27] also applied an experimental video-based research design to investigate the effect of physical disorder on American parents’ choices regarding their children’s walking routes. In addition to the different content of experimental manipulation, Nasar *et al*. [27] applied a different research design—a within-research design—in which each study participant was exposed to three study conditions. As always, different approaches have different strengths and weaknesses. We chose to apply a between-research design because we assumed that watching the six-minute video twice would lead to fatigue effects in our participants, which in turn might have an impact on the results of the study. 

The findings of the current study are not fully consistent with the results of previous studies. For example, a study of older adults in the Netherlands found no relation between the objectively measured presence of trees and walking for transportation [28]. Similarly, Li *et al*. [29] found no relationship between total green space and total walking of older adults. Rather, green strips were negatively related to the use of streets for recreational walking [28]. However, it has been shown that the presence of greenness and vegetation is related to the perceived attractiveness of streets for walking [30].

Thus, it may be that although (older) people prefer to walk on green streets, other factors moderate this effect. This assumption may be supported by the Belgian aging study, which investigated the relationships between physical environmental factors and both walking and cycling among older adults. Greenery was not associated with either walking or cycling by older adults for transportation or recreation in this study [31]. However, those people who feel insecure in their living environment showed lower volumes on walking for transportation as well as for walking for recreation. This apparent inconsistency seems to be an interesting issue. Our results suggest that the *combination* of parking spaces and green verges with trees affects older people’s perceptions of safety and their ratings of pedestrian friendliness. 

Nevertheless, these results must be interpreted with caution. Despite the methodological advancements in this study, the living environment was presented as a video. Thus, subjects took a “virtual trip” through a simulated living environment. The opportunity to walk through different real environments would be another promising advancement in the methodological approach to exploring the environmental mechanisms that influence feelings of safety and motivation to walk [20]. This approach would present an additional issue for research: What people notice in an environment depends on their walking speed [32]. 

The combination of parking spaces and green verges with trees in our study was a conscious decision. In Germany, it is common to combine parking spaces with green verges and trees. Therefore, our study was based on two methodological goals: first, we aimed to approximate subjects’ known reality to increase the ecological validity of the study, and second, we aimed to reduce the loss of internal validity by choosing a standardized experimental simulation. Parking spaces and grass verges with trees are micro-scale environmental characteristics that might be implemented when new living environments are planned and when existing sidewalks and streets are renovated. This study aims to provide evidence that might serve to improve decisions on which environmental changes could or should be realized. However, our results depend on the experimental setting and should be replicated in further studies. An experimental approach in the naturalistic setting would further increase ecological validity by including other factors, such as noise, smell, temperature or crowding. Those environmental stressors are also relevant to people’s behavior and health [33], and thus, are worth studying. 

In our study, the targeted population comprised older people without any functional limitations who live in urban environments. Our decision to limit participants to people 60 years of age or older is consistent with the cutoffs of the United Nations and World Health Organization. Another reason we excluded people younger than 60 years is that we wanted our study population to be retired. Although the legal retirement age in Germany is 65 years, the average actual retirement age is approximately 60 years (specifically, in 2010, the average age of retirement was 60.5 years for women and 61.1 years for men; www.statista.com).

Additionally, the subjects of this study reported a high level of habitual activity, with an average of more than 2.5 hours of moderate-to-vigorous physical activity (MVPA) per week. We assessed MVPA with a single global item, which might be an explanation for this. However, such a global single item has shown higher correlations with accelerometer data in validity studies than multi-item indices [34]. Comparable data from an average sample of their peers reveals that approximately 18% of Germans between the ages of 60 and 69 report that they get at least 2.5 hours of MVPA per week [35]. A recent review of international studies revealed that most studies report that between 20% and 60% of their respective samples get 2.5 hours of MVPA per week [36]. Although both, those and our results, suffer from over-reporting biases, we must note that our sample seemed more physically active in their daily lives than the average German sample. 

## 5. Conclusions 

Our findings support multiple conclusions. First, one of the basic assumptions of active living research is supported by our results: elements of the built environment might affect older people’s motivation to walk. Second, an experimental research design using dynamic computer simulation of built environments is feasible and appropriate in this field of research. Third, changes in the micro-environment had medium and strong effects on perceived traffic safety and pedestrian friendliness, respectively. The public health effect of such micro-environmental elements might be significant, assuming that improvements to these elements could lead to an increased walking time per person per week. Assuming that many people reside in cities with pedestrian-friendly living environments, the extrapolated public health effect could be substantial as even small increases in physical activity can lead to significant improvements in public health [37], especially in older people.
